# The genome sequence of *Glossophaga mutica* (Chiroptera, Phyllostomidae, Glossophaginae; Merriam, 1898)

**DOI:** 10.12688/wellcomeopenres.23611.1

**Published:** 2025-04-07

**Authors:** Nancy B. Simmons, Melissa R. Ingala, Brian P. O'Toole, Linelle Abueg, Kirsty McCaffrey, Bonhwang Koo, Giulio Formenti, Erich D. Jarvis, Myrtani Pieri, Meike Mai, Larry N. Singh, Philip Philge, Laramie L. Lindsey, Ning Zhang, Jonathan L. Gray, Emma C. Teeling, Sonja C. Vernes, the Bat Biology Foundation, The Bat1K Consortium

**Affiliations:** 1 Department of Mammalogy, Division of Vertebrate Zoology, American Museum of Natural History, New York NY, 10024, USA; 2 Department of Biological Sciences, Fairleigh Dickinson University, Madison, New Jersey, NJ 07940, USA; 3 Division of Mammals, Department of Vertebrate Zoology, National Museum of Natural History, Smithsonian Institution,, Washington, DC 20560, USA; 4 Department of Mammalogy, Division of Vertebrate Zoology, American Museum of Natural History, New York, NY 10024, USA; 5 Paratus Sciences, New York, USA; 6 Vertebrate Genome Lab, The Rockefeller University, New York, USA; 7 Excelra, Hyderabad, India; 8 Department of Life Sciences, School of Life and Health Sciences, University of Nicosia, Nicosia, Cyprus; 9 School of Biology, University of St Andrews, St Andrews, UK; 10 School of Biology and Environmental Science, University College Dublin, Dublin, Ireland; 11 Wellcome Sanger Institute, Wellcome Genome Campus, Cambridgeshire, CB10 1SA, UK

**Keywords:** Glossophaga mutica, genome sequence, chromosomal, Bat1K

## Abstract

We present a genome assembly from an individual female *Glossophaga mutica* (Chordata; Mammalia; Chiroptera; Phyllostomidae). The genome sequence is 2.13 in span. The majority of the assembly is scaffolded into 32 chromosomal pseudomolecules, with the X sex chromosome assembled.

## Species taxonomy

Eukaryota; Metazoa; Chordata; Craniata; Vertebrata; Euteleostomi; Mammalia; Eutheria; Laurasiatheria; Chiroptera; Yangochiroptera; Phyllostomidae; Glossophaginae, Glossophagini, *Glossophaga mutica* ^1– 3^.

## Introduction

**Figure 1.**
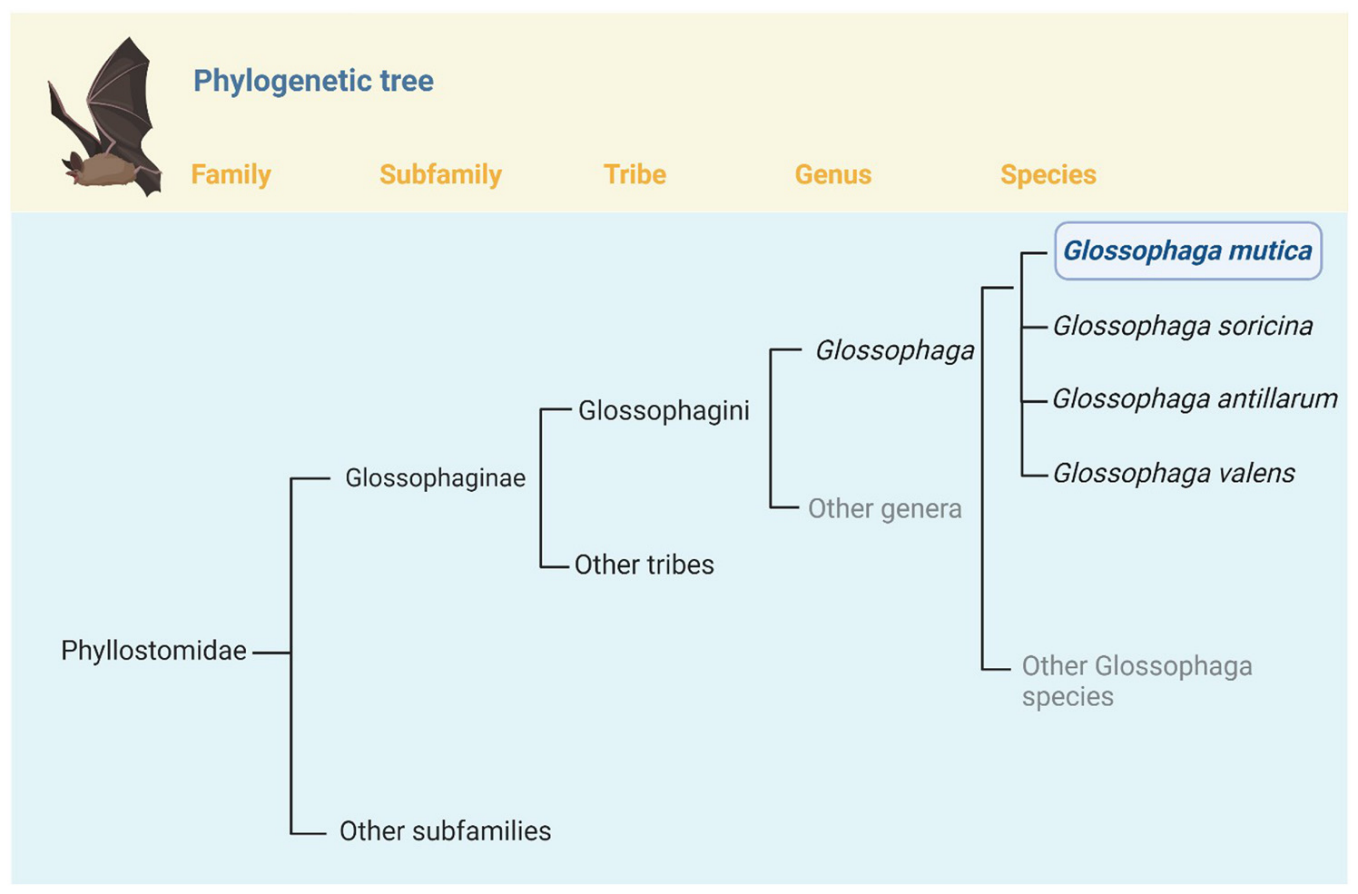
Position of *Glossophaga mutica* in the phylogeny of Family Phyllostomidae. *Glossophaga mutica* is one of 9 species currently recognized in the genus *Glossophaga* ^3^. *Glossophaga* belongs to the Tribe Glossophagini, which currently includes 3 genera and 14 species ^1– 3^. Within *Glossophaga*, the closest relatives of *G. mutica* are *G. soricina, G. antillarum*, and *G. valens*; until recently these taxa were considered conspecific ^19^. Phylogeny based on a variety of sources ^4, 19, 25– 27^.

The genus Glossophaga is a clade of small bodied nectarivorous/omnivorous bats belonging to the Tribe Glossophagini within the Subfamily Glossophaginae ^1– 3^ ( Figure 1). *Glossophaga* bats are common throughout the Neotropics with multiple species often occurring in sympatry ^4– 8^. Although *Glossophaga* species are frequently categorized as nectarivores because of their long rostrum and specialized tongue morphology (described by 9– 11), it has long been clear that many or all *Glossophaga* are actually facultative omnivores, variously feeding on nectar, pollen, fruit, and insects depending on the season and location ^5– 7, 12– 14^.

*Glossophaga mutica* Merriam, 1989 ^15^ is a member of the *Glossophaga soricina* species complex. Until recently, *mutica* was recognized as a subspecies of *G. soricina* ^4, 6, 8, 16– 18^. However, Calahorra-Oliart *et al.* ^19^ argued based on morphological and molecular evidence that *mutica* represents a species distinct from *soricina*, and this arrangement has been followed in the most recent comprehensive bat classifications ^3, 20^. As now defined, *Glossophaga mutica* (including populations formerly included in *G. soricina handleyi* Webster and Jones, 1980) ^21^ occurs in Mexico including the Tres Marias Islands, Guatemala, Belize, Honduras, El Salvador, Nicaragua, Costa Rica, Panama, and northern and western Colombia, while *G. soricina* is restricted to South America east of the Andes ^19, 20^. Calahorra-Oliart *et al.* ^20^ suggested that the mainland and Tres Marias islands forms of *G. mutica* should be considered different subspecies; in this case, mainland Central American populations would be called *G. mutica handleyi* Webster and Jones, 1980 ^21^ and the island populations would be referred to the nominate subspecies *G. mutica mutica*.

**Figure 2.**
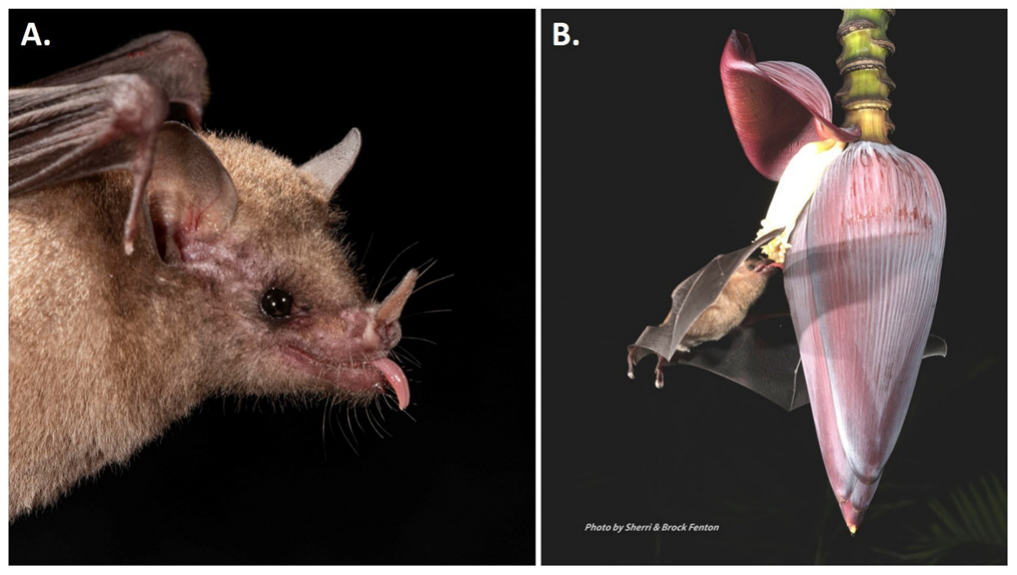
*Glossophaga mutica*. Adult individuals of *Glossophaga mutica*. **A**, portrait showing ear, face, and noseleaf morphology; note that the tongue is often extended over the chin in these bats. **B**, *Glossophaga mutica* feeding from a banana flower [Photos taken at Lamanai, Belize by Brock and Sherri Fenton].

*Glossophaga mutica* can be distinguished from congeners on the basis of morphological traits including size (forearm 33–38mm; greatest length of skull 19.9–21.9 mm; 7–15 g), dorsal fur brown (not dark brown) and bicolored with the base of hairs whitish; braincase relatively round; rostrum moderately long and of intermediate width; upper incisors almost equal in size and procumbent with tip of I1 protruding well beyond the tip of I2; lower incisors in contact with one another and canines with no gaps between adjacent teeth ^6, 7, 16, 19^. As shown in Figure 2, the tip of the tongue often extends out of the mouth when the bat is at rest.

Reports of the ecology and biology of *Glossophaga “soricina”* in Mexico and Central America apply to what is now known as *G. mutica*. This species is widespread and often abundant in the lowlands, especially in seasonally dry forest and disturbed areas; it is less common in evergreen forests and highland areas ^7^. It roosts in small to large groups in hollow trees, caves, tunnels, and anthropogenic structures including buildings and culverts ^7, 22^. The diet of *G. mutica* is diverse and consists of primarily moths and fruit in the wet season, and nectar and pollen in the dry season, but also includes beetles, dipterans, and leafhoppers ^7, 14, 23, 24^.

*Glossophaga mutica* has not been evaluated by the IUCN Redlist but was included in *Glossophaga soricina* (categorized as Least Concern) in the most recent evaluation by Barquez *et al.* ^28^. Given that *G. mutica* has a broad geographic range, is often abundant where it occurs, utilizes multiple habitats and roost types, and is not subject to any particular known threats, it is unlikely that it would be considered a threatened species.

## Genome sequence report

The genome was sequenced from a single female *Glossophaga mutica* (field number BZ-383, catalog number AMNH:Mammalogy:280902 collected at the Lamanai Archaeological Reserve, Orange Walk District, Belize, on 11 November 2021. A total of 46-fold coverage in Pacific Biosciences Hi-Fi long reads (contig N50 77 Mb) was generated after removal of all reads shorter than 10kb. Primary assembly contigs were scaffolded with chromosome confirmation Hi-C data. The final assembly has a total length of 2.13 Gb in 104 sequence scaffolds with a scaffold N50 of 170 Mb ( Table 1). The majority, 99.86%, of the assembly sequence was assigned to 32 chromosomal-level scaffolds, representing 15 autosomes (numbered by sequence length, and the X sex chromosome. Chromosomal pseudomolecules in the genome assembly of Glossophaga mutica are shown in Table 2. The assembly has a BUSCO ^29^ completeness of 96.6% using the laurasiatheria reference set.

**Table 1.** Genome data for *Glossophaga mutica.*

*Project accession data*	
Assembly identifier	GCA_039655065.1
Species	*Glossophaga mutica*
Specimen	mGloMut1
NCBI taxonomy ID	2994998
BioProject	Bat1K: Accession: PRJNA489245; ID: 489245
BioSample ID	SAMN40946060
Isolate information	Female – liver tissue
*Raw data accessions*	
Pacific Biosciences SEQUEL II	SRR29061521, SRR29061519, SRR29061517 (BAM); SRR29061522, SRR29061520, SRR29061518 (FASTQ)
Hi-C Illumina	SRR29061523, SRR29061524
Assembly accession	GCA_039655065.1
Accession of Alternative haplotype	GCA_039656995.1 (haplotype 2)
Span (Gb)	2.16
Number of contigs	219
Contig N50 length (Mb)	77
Number of scaffolds	82
Scaffold N50 length (Mb)	170
Longest scaffold (Mb)	228.4

* BUSCO scores based on the laurasiatheria_odb10set using v5.0.0. C= complete [S= single copy, D=duplicated], F=fragmented, M=missing, n=number of orthologues in comparison. * *Glossophaga mutica* BUSCO scores based on laurasiatheria_odb10 BUSCO set v5.3.2.

**Table 2.** Chromosomal pseudomolecules in the genome assembly of *Glossophaga mutica*. NCBI accession Chromosome Size (Mb) GC%. The chromosome number of *Glossophaga mutica* is 2n=32.

NCBI accession	Chromosome	Size (Mbp)	GC%
CM077303.1	1	224.30	0.4063
CM077304.1	2	223.83	0.4073
CM077305.1	3	219.48	0.4147
CM077306.1	4	213.30	0.4009
CM077307.1	5	177.83	0.4285
CM077308.1	6	169.73	0.4236
CM077309.1	7	143.34	0.4177
CM077310.1	8	133.53	0.4196
CM077318.1	X	116.65	0.4016
CM077311.1	9	115.37	0.4526
CM077312.1	10	108.76	0.4308
CM077313.1	11	97.23	0.4136
CM077314.1	12	66.63	0.4631
CM077315.1	13	58.47	0.4722
CM077316.1	14	54.22	0.4634
CM077317.1	15	22.81	0.5043

## Methods

The *Glossophaga mutica* specimen was a female individual collected on an American Museum of Natural History (AMNH) field expedition at the Lamanai Archaeological Reserve in the Orange Walk District of Belize. The individual sampled was identified as *Glossophaga mutica* based on morphometrics (e.g., forearm length, body mass) and morphological traits (e.g., rostrum proportions, pelage, and incisor morphology) as described above. The bat was caught in a 2-panel harp trap set on a trail near the Mask Temple in the Lamanai Archaeological Reserve (17.76715 N, 88.65135 W). All efforts were made to minimize any distress or suffering by the animal. The individual sampled was subjected to minimal handling after capture, and it was held in a clean cloth bag after capture as per best practices for field containment of bats. After species identification, the individual was euthanized humanely the same night it was captured. The animal was euthanized by isoflurane inhalation (<1ml to moisten cotton ball, formula CHF _2_OCCIHCF _3_), a humane approved method that rapidly causes unconsciousness and eventually death upon inhalation. Bats euthanized by this method are rendered unconscious within seconds due to their high respiration rate, and death occurs within a minute or two with no significant suffering by the animal. Capture and sampling were conducted under Belize Forest Department Permit FD/WL/1/21(16) and Belize Institute of Archaeology Permit IA/S/5/6/21(01), and samples were exported under Belize Forest Department permit FD/WL/7/22(07). All work was conducted with approval by the AMNH Institutional Animal Care and Use Committee (AMNHIACUC-20210614). All data were recorded and reported in accordance with the ARRIVE guidelines ^30^ – see data availability section and Table 1. Tissues were removed from the subject individual immediately following euthanasia and were flash-frozen in a liquid nitrogen dry shipper, with the cold chain maintained from field to museum to laboratory.

DNA was extracted using Nanobind extraction from muscle tissue following the Circulomics Nanobind HMW DNA Extraction Protocol. Pacific Biosciences HiFi libraries were constructed according to the manufacturer's instructions. Hi-C data was generated using the Arima Hi-C+ High Coverage kit from the liver tissue sample. Sequencing was performed by the Genomic Operations DNA Pipelines at Paratus Sciences on Pacific Biosciences Sequel IIe (HiFi reads) and Illumina NextSeq 2000 (Hi-C) instruments.

**Figure 3.**
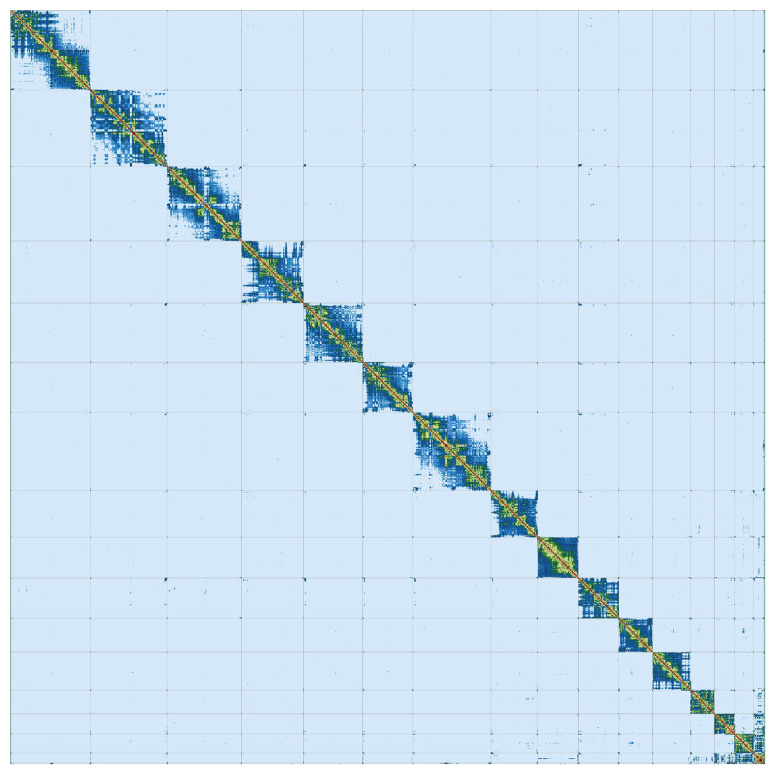
Hi-C Contact Map of the *Glossophaga mutica* assembly with *16* chromosomes, visualized using PretextView.

**Figure 4.**
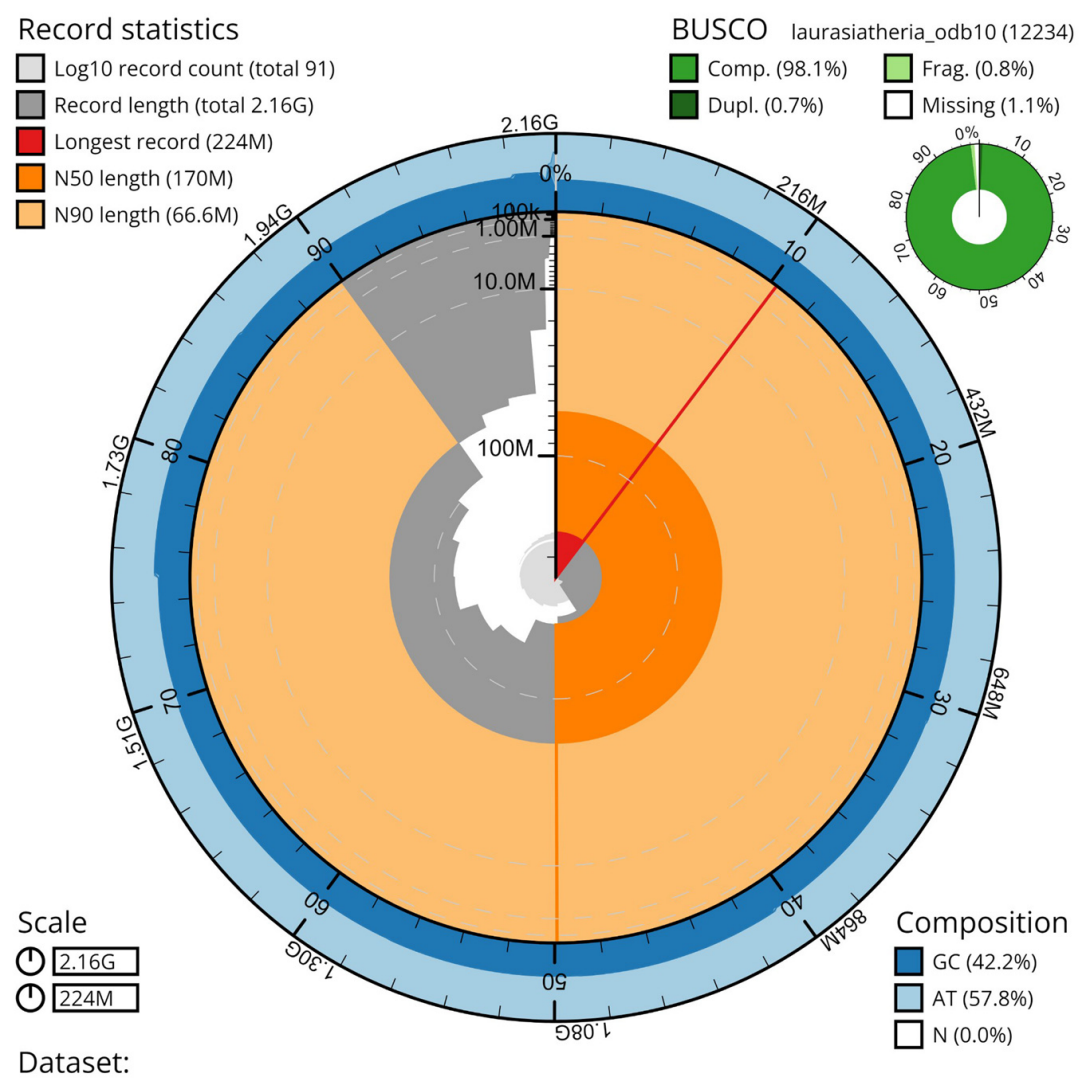
Genome assembly metrics generated using blobtoolkit for the *Glossophaga mutica* genome assembly. The larger snail plot depicts scaffold statistics including N50 length (bright orange) and base composition (blue). The smaller plot shows BUSCO completeness in green.

Assembly was carried out following the Vertebrate Genome Project Galaxy pipeline v2.0 ^31^. A brief synopsis of the method is as follows: Genome size was estimated using GenomeScope2 ^32^. Hifiasm with Hi-C phasing was used for genome assembly ^33^. The quality of the assembly was evaluated using Merqury ^34^ and BUSCO ^29^. Scaffolding with Hi-C data ^35^ was carried out with YaHS ^36^. PretextView was implemented to generate a Hi-C contact map ( Figure 3). Figure 4– Figure 6 were generated using BlobToolKit ^37^. Software utilised for the *G. mutica* analysis are depicted in Table 3.

**Figure 5.**
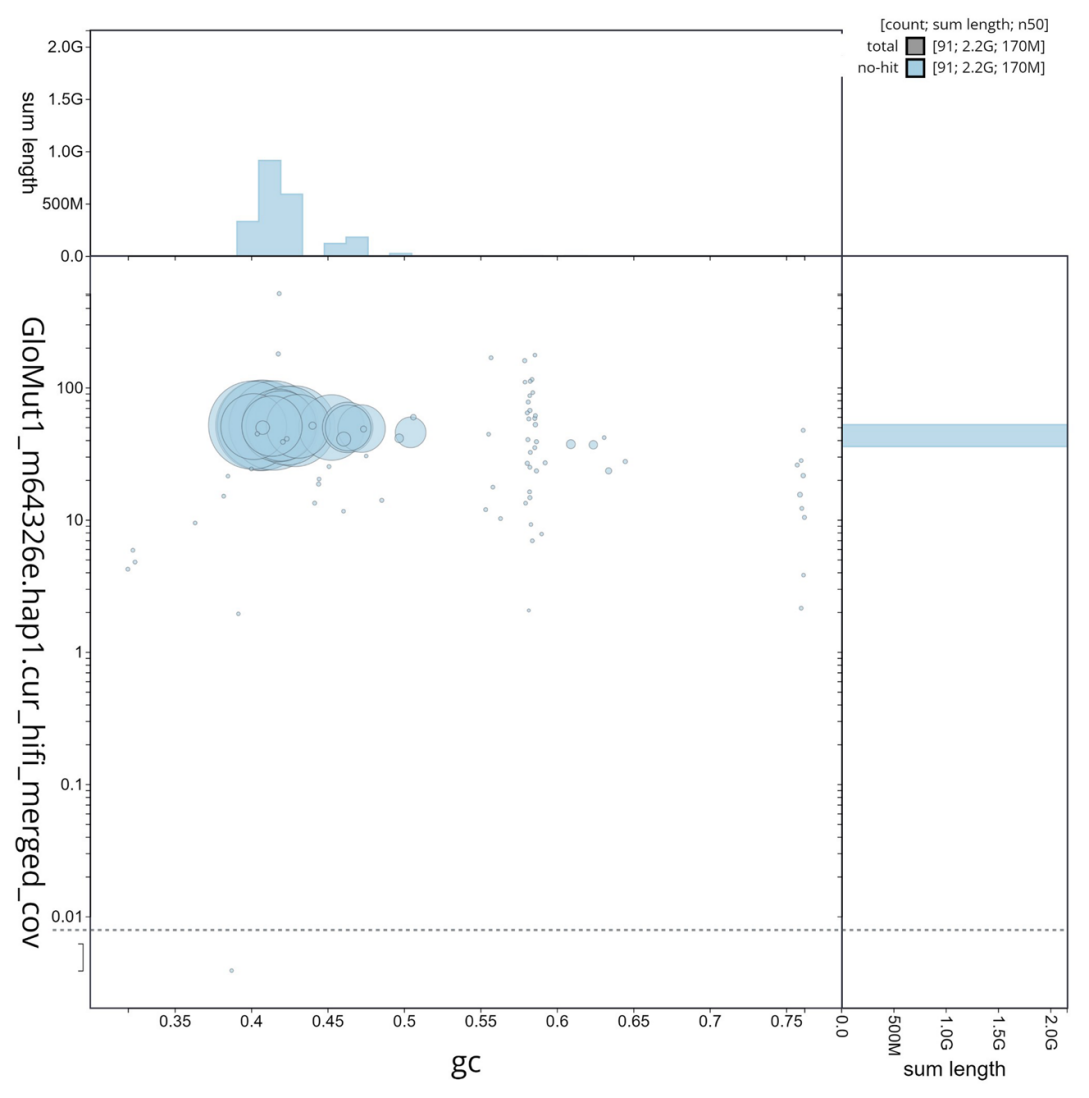
GC coverage plot generated for the *Glossophaga mutica* assembly using blobtoolkit. Individual chromosomes and scaffolds are represented by each circle. The circles are sized in proportion to chromosome/scaffold length. Histograms show the sum length of chromosome/scaffold size along each axis. Color of circles indicate taxonomic hits of each Phylum represented in the assembly.

**Figure 6.**
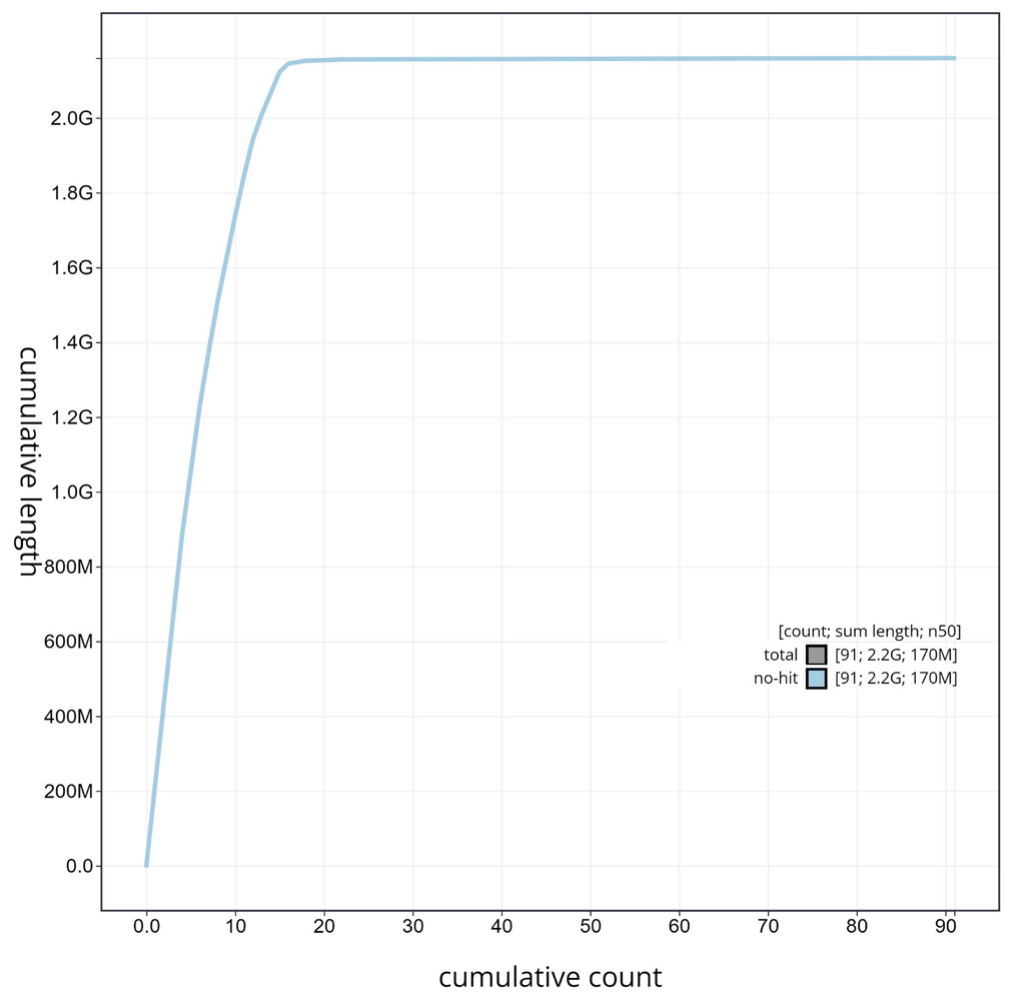
Cumulative sequence plot generated for the *Glossophaga mutica* assembly using blobtoolkit. The grey line shows the cumulative length for all chromosomes/scaffolds in the assembly. Colored lines represent Phylum represented in the assembly.

**Table 3.** Software tools used.

Software tool	Version	Source
bamUtil	1.0.15	https://genome.sph.umich.edu/wiki/BamUtil:_bam2FastQ
MultiQC	1.13	https://github.com/ewels/MultiQC
Genomescope	2.0	https://github.com/tbenavi1/genomescope2.0
hifiasm	0.19.3	https://github.com/chhylp123/hifiasm
purge_dups	1.2.6	https://github.com/dfguan/purge_dups
BUSCO	5.3.2	https://busco.ezlab.org/
Merqury	1.3	https://github.com/marbl/merqury
Assembly-stats	17.02	https://github.com/rjchallis/assembly-stats
Arima-HiC Mapping Pipeline	-	https://github.com/ArimaGenomics/mapping_pipeline
YaHS	1.1	https://github.com/c-zhou/yahs
HiGlass	1.11.7	https://github.com/higlass/higlass
samtools	1.9	https://www.htslib.org/
PretextView	-	https://github.com/sanger-tol/PretextView/tree/master
BUSCO	5.7.0	https://busco.ezlab.org/
BlobToolKit	4.3.5	https://github.com/blobtoolkit/blobtoolkit
pbmm2	1.13.1	https://github.com/PacificBiosciences/pbmm2
Blast	2.15.0+	https://blast.ncbi.nlm.nih.gov/Blast.cgi

## Ethics

All work was conducted with approval by the AMNH Institutional Animal Care and Use Committee (AMNHIACUC-20210614 given on 6/14/2021). All efforts were made to ameliorate suffering of animals.

## Data Availability

The ***Glossophaga mutica*** genome sequencing initiative is part of the Bat1K genome sequencing project. The genome assembly is released openly for reuse. The genome assembly for *Glossophaga mutica* (Merriam's long-tongued bat) can be found in the NCBI and European Nucleotide Archive. The assembly accession number at NCBI is GCA_039655065.1, and more details can be accessed through this link: https://www.ncbi.nlm.nih.gov/datasets/genome/GCA_039655065.1/ ^38^. The genome assembly can be found in the European Nucleotide Archive: *Glossophaga mutica* (Merriam's long-tongued bat). Accession number JBCAQL000000000 (haplotype 1), https://www.ebi.ac.uk/ena/browser/view/JBCAQL000000000; JBCAQM000000000 (haplotype 2), https://www.ebi.ac.uk/ena/browser/view/JBCAQM000000000 ^39^ All raw sequence data and the assembly have been deposited in NCBI (PRJNA1101976). Data accession identifiers are reported in Table 1. Zenodo : ARRIVE checklist for “The genome sequence of *Glossophaga mutica* (Chiroptera, Phyllostomidae, Glossophaginae; Merriam, 1898)” https://doi.org/10.5281/zenodo.14627905 ^40^ Data are available under the terms of the Creative Commons Attribution 4.0 International license (CC-BY 4.0).
