# Supplementary material for: The genome sequence of Glossophaga mutica (Chiroptera, Phyllostomidae, Glossophaginae; Merriam, 1898)
Source: Wellcome Open Res. 2025 Apr 7;10:174. [Version 1] doi: 10.12688/wellcomeopenres.23611.1 (PMC12082079; doi:10.12688/wellcomeopenres.23611.1)
Supplement: Peer review history

# Reviewer report: Reviewer response for version 1

**DOI:** 10.21956/wellcomeopenres.26047.r122045

**Published:** 2025-05-26

**Authors:** Balaji Chattopadhyay

**Affiliations:** 1 Ashoka University, Haryana, India

The authors report a high quality genome assembly of a female individual of Glossophaga mutica.

 The authors have provided necessary information of the species taxonomy and ecology. The methods and results contain sufficient details as a genome sequence report. While not necessary, the authors may consider adding details of the extent of sequences generated.

Are sufficient details of methods and materials provided to allow replication by others?

Yes

Is the rationale for creating the dataset(s) clearly described?

Yes

Are the datasets clearly presented in a useable and accessible format?

Yes

Are the protocols appropriate and is the work technically sound?

Yes

Reviewer Expertise:

genomics, bats, evolution, biodiversity genomics

I confirm that I have read this submission and believe that I have an appropriate level of expertise to confirm that it is of an acceptable scientific standard.

# Reviewer report: Reviewer response for version 1

**DOI:** 10.21956/wellcomeopenres.26047.r122050

**Published:** 2025-05-13

**Authors:** Thiago Bernardi Vieira

**Affiliations:** 1 Universidade Federal do Pará, Belém - PA, Brazil

Dears,

 I finished reading *The Genome Sequence of Glossophaga mutica* (Chiroptera, Phyllostomidae, Glossophaginae; Merriam, 1898) and believe it is excellent work. The authors begin with a historical description of the species and its geographic range. Although this study focuses on genomic characterization—and other works already provide distribution maps—the authors might consider including a map with the geographic delimitations of the species. However, this is not mandatory.

 Regarding the genomic DNA extraction, I found it slightly difficult to understand how the DNA was extracted from muscle tissue. This might be due to my limited familiarity with DNA extraction protocols.

 Overall, the article is well-written and a pleasure to read and the data are well presented.

 Best regards,

Are sufficient details of methods and materials provided to allow replication by others?

Partly

Is the rationale for creating the dataset(s) clearly described?

Yes

Are the datasets clearly presented in a useable and accessible format?

Yes

Are the protocols appropriate and is the work technically sound?

Yes

Reviewer Expertise:

Ecology and conservation of bats, community and metacommunity analysis, seed dispersal, and bat-fly network interactions.

I confirm that I have read this submission and believe that I have an appropriate level of expertise to confirm that it is of an acceptable scientific standard.

# Reviewer report: Reviewer response for version 1

**DOI:** 10.21956/wellcomeopenres.26047.r122047

**Published:** 2025-04-18

**Authors:** Guilherme Siniciato Terra Garbino

**Affiliations:** 1 Museu de Zoologia João Moojen, Departamento de Biologia Animal, Federal University of Vicosa, Viçosa, State of Minas Gerais, Brazil

This article presents a genome assembly of Glossophaga mutica, a species that was part of the once widespread Glossophaga soricina.

 The article is straighforward and clearly describe the methods used, while also giving a comprehensive overview on the species, including a bit of its taxonomic history.

 I have just a few very minor comments:

 On the first line of the Introduction, please italicize "Glossophaga" in "The genus Glossophaga..". Also italicize the species name in "Chromosomal pseudomolecules in the genome assembly of Glossophaga mutica" [genome sequence report section]

 In "evaluated by the IUCN Redlist", "Redlist" should be separated, as "Red List"

 In the "Genome sequence report" section, the parenthesis that begins in "(field number BZ-383, catalog number..." need to end in ".. November 2021." - or perhaps it should end after "AMNH:Mammalogy:280902" ?

 It is not clear why this reference is cited in the text, as it apparently is not related to the subject investigated: Ref :1

Are sufficient details of methods and materials provided to allow replication by others?

Yes

Is the rationale for creating the dataset(s) clearly described?

Yes

Are the datasets clearly presented in a useable and accessible format?

Yes

Are the protocols appropriate and is the work technically sound?

Yes

Reviewer Expertise:

Systematics, biogeography, phylogenetic

I confirm that I have read this submission and believe that I have an appropriate level of expertise to confirm that it is of an acceptable scientific standard.

## References

[1] : D. E. Wilson and R. A. Mittermeier (chief editors): Handbook of the Mammals of the World. Vol. 9. Bats. *Mammalian Biology* .2020;100(3) : 10.1007/s42991-020-00026-w 10.1007/s42991-020-00026-w
